# Subtypes of minimal residual disease, association with Gleason score, risk and time to biochemical failure in pT2 prostate cancer treated with radical prostatectomy

**DOI:** 10.3332/ecancer.2019.934

**Published:** 2019-06-06

**Authors:** Nigel P Murray, Socrates Aedo, Cynthia Fuentealba, Eduardo Reyes, Anibal Salazar, Marco Antonio Lopez, Simona Minzer, Shenda Orrego, Eghon Guzman

**Affiliations:** 1 Faculty of Medicine, University Finis Terrae, Pedro de Valdivia 1509, Providencia, Santiago 7501015, Chile; 2 Urology Service, Hospital de Carabineros, Simón Bolívar 2200, Ñuñoa, Santiago 7770199, Chile; 3 Faculty of Medicine, University Diego Portales, Manuel Rodríguez Sur 415, Santiago 8370179, Chile; 4 Urology Service, Hospital DIPRECA, Vital Apoquindo 1200, Las Condes, Santiago 7601003, Chile; 5 Faculty of Medicine, University Mayor, San Pio X 2422, Providencia, Santiago 7510041, Chile

**Keywords:** prostate cancer, biochemical failure, minimal residual disease, circulating tumour cells, micro-metastasis

## Abstract

**Introduction:**

The Gleason score is a strong prognostic factor for treatment failure in pathologically organ-confined prostate cancer (pT2) treated by radical prostatectomy (RP). However, within each Gleason score, there is clinical heterogeneity with respect to treatment outcome, even in patients with the same pathological stage and prostate-specific antigen (PSA) at diagnosis. This may be due to minimal residual disease (MRD) remaining after surgery. We hypothesise that the sub-type of MRD determines the risk of and timing of treatment failure, is a biological classification, and may explain in part clinical heterogeneity. We present a study of pT2 patients treated with RP, the subtypes of MRD for each Gleason score and clinical outcomes.

**Patients and methods:**

Patients with Gleason ≤6 (G6) or Gleason 7 (G7) pT2 cancer participated in the study. One month after surgery, blood was taken for circulating prostate cell (CPCs); mononuclear cells were obtained by differential gel centrifugation and identified using immunocytochemistry with anti-PSA. The detection of one CPC/sample was defined as a positive test. Touch-preparations from bone-marrow biopsies were used to detect micro-metastasis using immunocytochemistry with anti-PSA. Biochemical failure was defined as a PSA >0.2 ng/mL. Patients were classified as: Group A MRD negative (CPC and micro-metastasis negative), Group B (only micro-metastasis positive) and Group C (CPC positive). Biochemical failure-free survival (BFFS) using Kaplan–Meier and time to failure using Restricted Mean Survival Time (RMST) after 10 years of follow-up were calculated for each group based on the Gleason score.

**Results:**

Of a cohort of 253 men, four were excluded for having Gleason 8 or 9 prostate cancer, leaving a study group of 249 men of whom 52 had G7 prostate cancer. G7 patients had a higher frequency of MRD (69% versus 36%) and worse prognosis. G6 and G7 patients negative for MRD had similar BBFS rates, 98% at 10 years, time to failure 9.9 years. Group C, G6 patients had a higher BFFS and longer time to failure compared to G7 patients (19% versus 5% and 7 versus 3 years). Group B showed similar results up to 5 years, thereafter G6 had a lower BFFS 63% versus 90%.

**Conclusions:**

G7 and G6 pT2 patients have different patterns of MRD and relapse. Risk stratification using MRD sub-types may help to define the need for adjuvant therapy. This needs confirmation with large randomised long-term trials.

## Introduction

The use of prostate-specific antigen (PSA) as a screening test has resulted in a migration to earlier stage cancer, with the majority of men being diagnosed with non-palpable, clinically localised disease [1, 2]. Although the percentage of patients with pathologically organ-confined tumours has substantially increased [3], 4%–32% of these men will eventually relapse following radical prostatectomy (RP) [4–6]. Multivariate analysis has reported that the pathological tumour grade (Gleason score) and pre-operative serum PSA are highly predictive of outcome following RP for pathologically localised PC [7].

In patients who achieve a PSA nadir of <0.01 ng/mL post-surgery, the failure of curative surgery is hard to explain. Although the peak time to relapse is 2 years, the majority will do so within 5 years [8, 9], but many patients remain clinically disease-free for years until there is an increase in the serum PSA or overt metastasis are detected. Although an erroneous pathological classification of the tumour, in terms of either the cancer penetrating the prostate capsule (pT3) or an anatomically incorrect dissection plane (unrevealed positive margin), which left behind microscopic amounts of PC, which then subsequently progressed, may explain some cases, this is not the case in the majority. The presence of sub-clinical micro-metastasis not detected by conventional imaging is a more logical explanation of these cases. These microscopic foci not removed by radical surgery are termed minimal residual disease (MRD).

Early in the disease process, tumour cells disseminate first to the neuro-vascular structures and then into the circulation [10]. It has been estimated that approximately 10^6^ circulating prostate cells (CPCs) per gram of primary tumour are released into the circulation daily [11]. However, most of these CPCs will not survive, being destroyed by shear forces within the circulation or not having the phenotypic characteristics to implant and survive in distant tissues. Data derived from animal models have reported that less than 0.01% of tumour cells that enter the bloodstream have the ability to form a single bony metastasis [12]. In prostate cancer, the bone marrow is the most common site involved by metastatic tumours. However, tumour cells may infiltrate the bone marrow without any abnormalities recognised in conventional imaging studies, bone scans, serum biochemistry and/or haematological parameters. Prostate cancer cells have been found to be present in the bone marrow in between 13% and 72% of prostate cancer patients prior to RP [13, 14].

These tumour cells may remain latent for prolonged periods of time [15] and in clinical practice; this is seen as a prolonged disease-free survival between the removal of the primary tumour and disease recurrence. Due to changes in the tumour cells or the stromal microenvironment, these latent or quiescent cells enter a proliferative phase and re-enter the circulation where they are detected as CPCs. Their presence is reported to be associated with a worse prognosis and early failure [16, 17]. The detection of bone marrow micro-metastasis has given conflicting results and may be associated with late relapse [16, 18]. More recently, it has been reported that the detection of CPCs is associated with increased, early biochemical failure, independent of the presence or absence of bone marrow micro-metastasis, whereas those men with only bone micro-metastasis are at risk of late relapse [19].

To explain these differences in treatment failure, we hypothesise that the clinical heterogeneity of Gleason 6 and 7 patients is a consequence of biological differences within each Gleason score, these differences are reflected in different subtypes of MRD. These sub-types of MRD have differing risks and time to treatment failure, given that the Gleason score is such a strong predictor of prognosis identifying subgroups of MRD could improve risk stratification and treatment selection.

The objective of this study was to determine the presence of CPCs and micro-metastasis in men with pathologically organ-confined prostate cancer treated with RP as mono-therapy and to determine the association of the sub-type of MRD with the Gleason score and with the risk and time to treatment failure.

## Patients and methods

This was a single centre, prospective observational study of men who underwent RP as mono-therapy for prostate cancer. Consecutive patients undergoing RP for prostate cancer were invited to participate. Patient recruitment was from January 2000 to December 2005 and follow-up was continued until biochemical failure or last control until December 2015, the end of the study period.

Clinical details of age and pre-treatment serum total PSA were measured before the digital rectal examination using the Siemens Advia CentaurXR® assay. The surgical sample was analysed to determine the Gleason score and pathological stage and 1 month after surgery blood, and bone marrow samples were taken for the detection of CPCs and bone marrow micro-metastasis.

### Pathological study

The pathological study of the surgical piece was performed by dedicated genitourinary pathologists according to the Gleason system. Pathological stage was defined according to the Partin criteria, organ confined, extracapsular extension, seminal vesicle invasion and lymph node invasion [20].

All men had a nadir PSA post-surgery of <0.01 ng/mL.

### Inclusion criteria

1. Pathologically localised pT2 prostate cancer treated with RP; defined according to the Partin criteria.

### Exclusion criteria

1. Patients with extra-capsular extension; defined as cancer cells in contact with the prostatic capsule.
2. Patients with a positive surgical margin; defined as cancer cells in contact with the inked surface of the surgical specimen.
3. Previous treatment or consideration for treatment with androgen blockade.
4. Consideration for adjuvant radiotherapy.
5. Infiltration of the seminal vesicles and/or regional lymph nodes with cancer.
6. Men with a positive bone scan were also excluded.

In accordance with the NCCN guidelines of 2000, pT2 prostate cancer was treated with monotherapy, in this study RP, no further treatment was recommended until treatment failure.

Patients were followed up with serial total PSA levels, three monthly for the first year and six monthly thereafter. Biochemical failure was defined as a serum PSA >0.2 ng/mL on two separate occasions. Biochemical failure-free survival time (BFFS) was defined as the time from surgery to the time of a post-surgery PSA of >0.20 ng/mL or last follow-up date.

### Detection of secondary circulating prostate cells

One-month post-surgery, an 8-mL venous blood sample was taken and collected in a tube containing ethylene-diamino-tetra-acetic acid (EDTA) (Vacutainer®, USA). Samples were maintained at 4°C and processed within 48 hours. CPC detection was independently evaluated with the evaluators being blinded to the clinical details.

### Collection of CPCs

Mononuclear cells were obtained by differential centrifugation using Histopaque 1,077 (Sigma-Aldrich, USA), washed and re-suspended in a 100 μL aliquot of autologous plasma. 25 μL aliquots were used to make slides (silanised, DAKO, USA), which were dried in air and fixed.

### Immunocytochemistry

**Figure 1.**
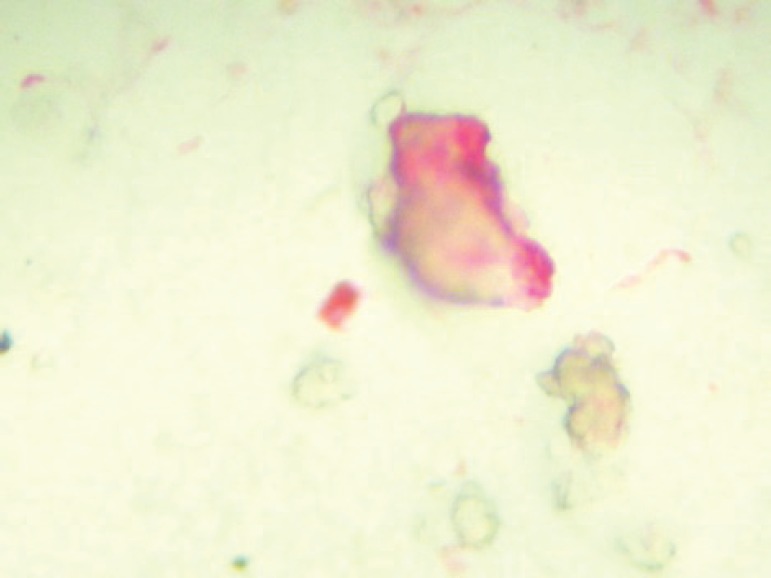
CPC staining red for PSA.

CPCs were detected using a monoclonal antibody directed against PSA, clone 28A4 (Novocastro Laboratory, UK), and identified using an alkaline phosphatase-anti-alkaline phosphatase based system (LSAB2, DAKO, USA), with new fuchsin as the chromogen. Samples positive for PSA staining cells underwent a second process. The slides were incubated with anti-CD45 clone 2B11 + PD7/26 (DAKO, USA) and cells identified with a peroxidase-based system (LSAB2, DAKO, USA) with DAB (3,3 diaminobenzidine tetrahydrochloride) as the chromogen. A secondary CPC was defined according to the criteria of International Society of Hemotherapy and Genetic Engineering [21] and as expressing PSA but not CD45 and a leukocyte as expressing CD45 but not PSA (Figure 1). A test was considered positive for secondary CPCs when at least 1 cell/8 mL of blood was detected.

### Bone marrow biopsy

Although previous studies have used bone marrow aspirates to detect micro-metastasis, we used biopsy specimens. We have previously reported that the prostate tumour cells detected in bone marrow aspirates are phenotypically different than those prostate cells detected in bone marrow biopsies and may not represent ‘true’ micro-metastasis but rather cells circulating within the bone marrow [22]). For this reason, bone marrow biopsy ‘touch preps’ was used as the sample to test for micro-metastasis.

**Figure 2.**
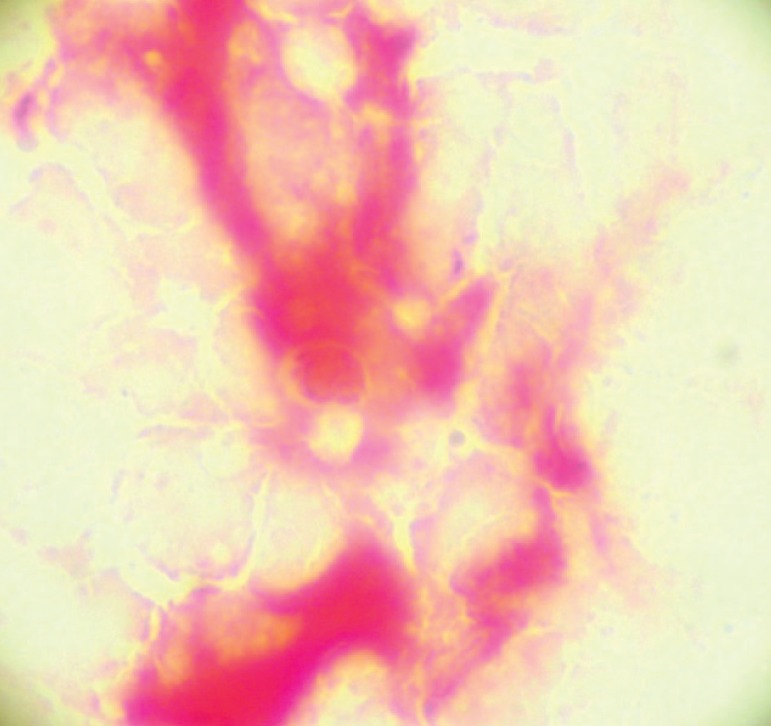
Bone marrow micro-metastasis staining red for PSA.

Bilateral bone marrow biopsies were taken from the posterior superior iliac crest 1 month after surgery and the sample used to prepare four ‘touch preps’ using salinised slides (DAKO, USA). All four slides were processed as described for CPCs, a micro-metastasis was defined as cells staining positive for PSA and negative for CD45 (Figure 2).

The patients were divided into three groups according to the presence or absence of CPCs and micro-metastasis; Group A was negative for both CPCs and micro-metastasis patients; Group B was CPC negative, micro-metastasis positive and Group C was CPC positive with or without bone marrow micro-metastasis detected.

### Study end point

The primary study end point was the presence of biochemical failure and secondary end point mean time to failure after the primary treatment.

### Statistical analysis

The analysis was performed using the program Stata (Stata/SE 15.0 for Windows, Copyright 1985-2017 StataCorp LLC) describing according to the nature and distribution of the quantitative and ordinate variables with measurements of central tendency (mean and median) and of dispersion using the inter-quartile range (IQR) and standard deviation [23]. The Shapiro–Wilk Test was used to define the null hypothesis with respect to the normal distribution. The nominal dichotomous variables were described as proportions with their respective confidence intervals [23].

The three prognostic groups were compared for age, total serum PSA and Gleason score of 7. Pearson’s chi-squared test was used to compare frequencies. A *p* value < 0.05 was taken to signify the statistical significance and all tests were two-tailed [23].

In the whole cohort and by prognostic group (groups A, B and C), a nonparametric survival analysis was performed at 3, 5 and 10 years of follow-up, establishing the survival proportion of Kaplan–Meier and median [23, 24]. The restricted mean survival time (RMST) establishes the expected time for an event to occur during a determined time period and its value is the area under the Kaplan-Meier non-parametric survival curve [24]. For each prognostic group the RMST was calculated for a period of ten years.

A parametric alternative to the Cox model, known as a flexible parametric survival model (FP model), allows us in a predictive way (not descriptive like Kaplan–Meier model) to determine the RMST and the hazard ratio when there is no compliance with the proportional risk assumptions (Cox model) [25–27].

The calibration aspect of the model refers to agreements between the predicted outcome and the observed outcome [28] and is shown graphically comparing predicted survival and observed survival (Kaplan–Meier). The discrimination of a prognostic model reflects its ability to distinguish between patient outcomes. We calculated: The Harrell’s C discrimination index [28], which is scored on a scale of 0 to 1. This can be taken to mean that if two cases are drawn at random, the C statistic is the probability that the person who survives the longest had the highest predicted survival. Values near 0.5 suggest the prognostic score is equivalent to a coin toss in determining which patient will live longer, while values near 0 or 1 indicate perfect discrimination.

From the FP model for biochemical failure to 10 years, the RMST were determined for each prognostic group and Gleason score greater than 6. The HR for each significant predictor of model based on values of the other significant predictors were calculated.

### Ethical considerations

The study was approved by the local ethics committee and in complete agreement with the Declaration of Helsinki. All the patients provided written informed consent.

## Results

A total of 253 men participated in the study, of which four patients had a Gleason score of 8 or 9 and excluded for the small number of patients. Thus, the total observed cohort included 249 subjects, whose follow-up time showed a median of 7.6 years (IQR: 5.3 years) with minimum and maximum, respectively, of 0.08 and 12 years.

The median age of the whole cohort was 66 years (IQR 12 years) and a median serum PSA at the time of diagnosis of 4.98 ng/mL (IQR 1.44 ng/m)L. Fifty-two (20.9%) men had a Gleason score of 7, 54 (21.69%) subjects were positive for CPCs and 85 (34.14%) positives for the presence of micro-metastasis.

The distribution by prognostic groups observed was 144 men (57.0%) group A, 53 men (21.3%) group B and 54 men (21.7%) group C. Table 1 shows the comparison between the prognostic groups for age, PSA and Gleason Score 7.

There were no significant differences with respect to age or serum PSA at the time of diagnosis between the different groups. However, Gleason 7 patients had a significantly higher frequency of MRD and CPC positive disease (Groups B and C) as compared to Gleason 6 patients. 127/197 (64.5%) of Gleason 6 patients were negative for micro-metastasis or CPCs, in comparison, only 15/52 (28.9%) of Gleason 7 patients were negative for micro-metastasis and/or CPCs (*p* < 0.001). In terms of CPC positivity, 21/52 (40.4%) patients with Gleason 7 cancer had CPCs detected in comparison with 33/197 (16.8%) of Gleason 6 patients (*p* < 0.0005).

**Figure 3.**
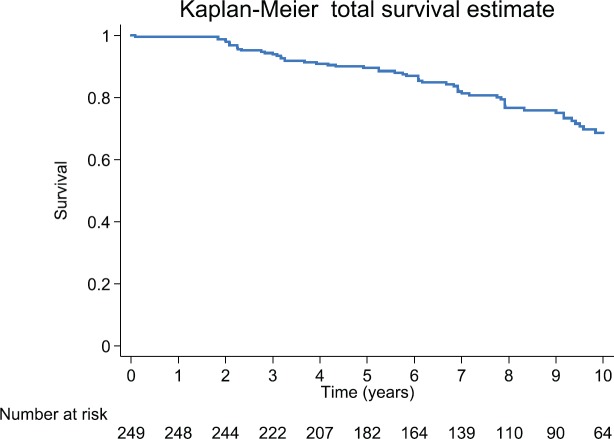
Observed survival (Kaplan–Meier) biochemical failure-free progression at 10 years by total cohort, prognostic groups and Gleason score of 7, on 249 men Treated by RP for prostate cancer.

After 3, 5 and 10 years of follow-up, the Kaplan–Meier survival for biochemical failure on the whole group was: 94.3% (95%CI: 90.6–96.6), 89.5 (95%CI: 84.8–92.8) and 68.6% (95%CI: 60.6–75.3), respectively. For the whole cohort, the observed survival median was not reached (Figure 3). The RMST (area under the Kaplan–Meier nonparametric survival curve) at 10 years for the whole study group was of 8.8 years (95%CI: 8.5–9.1).

**Figure 4.**
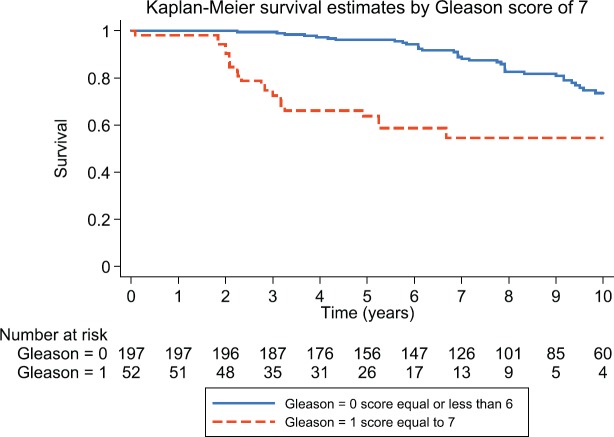
Kaplan–Meier survival estimates for patients with Gleason 6 and Gleason 7 prostate cancer.

After 3, 5 and 10 years of follow-up, the observed Kaplan–Meier survival for biochemical failure by Gleason score is shown in Figure 4, patients with a Gleason 7 score had worse biochemical failure-free survival than patients with Gleason 6 prostate cancer.

**Figure 5.**
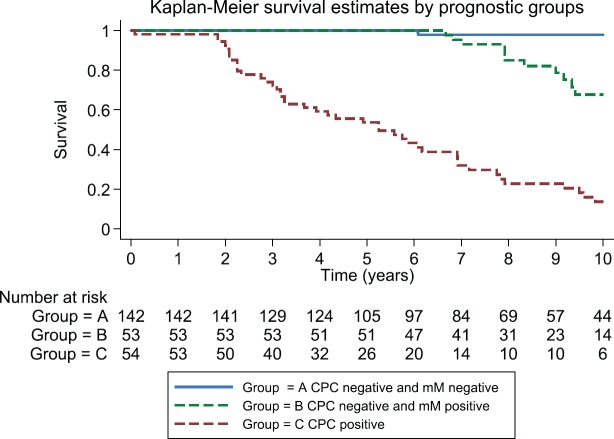
Kaplan–Meier survival estimates by prognostic groups at 10 years.

**Figure 6.**
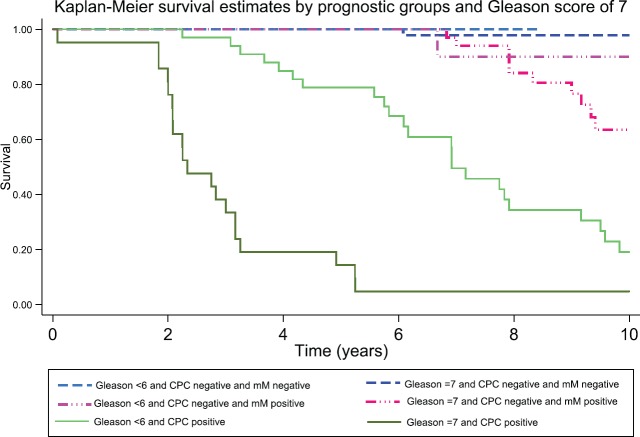
Kaplan–Meier survival estimates by prognostic group and Gleason score at 10 years.

After 3, 5 and 10 years of follow-up, the observed Kaplan–Meier survival for biochemical failure by the prognostic group and Gleason score are shown in Table 2 and Figures 5 and 6. The median survival was observed only for the prognostic group C, with a value of 5.25 years (95%CI: 3.25–6.92). The Log-Rank Test showed a *p*-value less than 0.01 at comparing the survival for biochemical failure between the prognostic groups.

Patients negative for both micro-metastasis and CPCs had the best prognosis; those with Gleason 6 tumours had a 98% biochemical failure-free survival at 10 years and those with Gleason 7 tumours 100% at 5 years (no Gleason 7 patients had a follow-up of more than 5 years in Group A). In Group B, progression-free survival in both Gleason 6 and 7 patients was similar to those to Group A for the first 5 years, thereafter there was an increasing biochemical failure, being higher in Gleason 7 patients (63% disease-free progression versus 90% disease-free progression). Group C patients had a significantly worse prognosis with a high frequency of early failure, patients with Gleason 7 tumours having a significantly worse prognosis than those with Gleason 6 tumours (5% progression-free survival versus 19% at 10 years, respectively).

Table 3 shows the RMST, Group A had the mean longest time to disease progression as compared with Groups B and C, in Group A, Gleason 6 patients had a longer time to failure than those with Gleason 7 tumours. Group B patients had an intermediate mean time to failure; those with Gleason 7 tumours had a significantly shorter mean time to failure than Gleason 6 patients. Group C had the shortest mean time to disease progression; those with Gleason 7 tumours had a significantly shorter time to failure than Gleason 6 patients.

**Figure 7.**
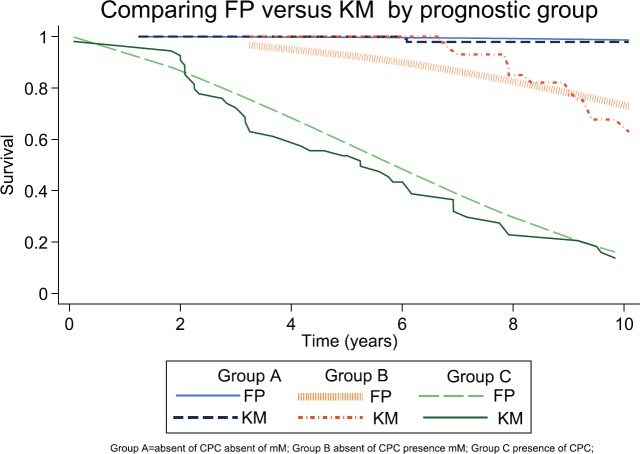
Comparing Kaplan–Meier observed survival estimates versus FP Model predicted survival estimates for prognostic group.

The predicted FP survival model was used to calculate the baseline hazard rate, (Gleason 6 and Group A); the hazard rates for biochemical failure were Group B 3.08 (*p* < 0.01), Group C 4.91 (*p* < 0.01) and Gleason 7 4.91 (*p* < 0.01). There was agreement when comparing the predicted FP model with the observed survival (Kaplan–Meier Survival) (Figures 3 and 7; Table 2). Harrell’s C discrimination index for the predicted FP model was 0.92, which is classified as very good.

The predicted FP model showed that the patients with a Gleason score of 7 had a decreasing failure risk with time, its observed values going from 46.69 at 6 months until 0.92 at 10 years. In contrast, the predicted FP model showed that patients with a Gleason score of 6 had a constant risk of biochemical failure with time.

## Discussion

In pT2 prostate cancer treated with RP, the entire tumour has theoretically been surgically removed. However, a significant number of patients will experience treatment failure within 10 years. Gleason score and pre-treatment PSA levels are important prognostic factors in pT2 cancer and are used to classify the risk of relapse. 15-year prostate cancer-specific mortality after RP is predicted by primary and secondary Gleason score, seminal vesicle invasion and year of surgery, with increasing mortality rate with increasing Gleason score [29]. Even in the early stages, prostate cancer is a systemic disease [10], however the fate of tumour cells which implant in distant sites such as bone will depend on the interactions of the tumour cell with the microenvironment. The ability of cancer cells to survive, proliferate, induce angiogenesis and invasion beyond the bone marrow was based on the ‘soil and seed’ theory of Paget and later modified by Mundy *et al* [30], with a concept known as the ‘vicious cycle’ whereby cancer cells once established in bone modify their immediate environment to support their own survival and growth. The balance between immunological factors in the microenvironment and phenotypic characteristics of the tumour cells will decide the outcome [31]. These characteristics may change with time; the bone marrow microenvironment is not passive and can attract and react to infiltrating tumour cells [32, 33]. Similarly, tumour cells are heterogeneous, are highly plastic in their phenotypic characteristics and may change from a latent/quiescent state to one of reactivation and proliferation, which is clinically seen as a relapse many years after primary curative treatment [34].

In this reported study, pT2 patients were divided into three groups, those patients negative for micro-metastasis and CPCs (Group A), those patients positive for only micro-metastasis (Group B) and those positive for CPCs independent of whether there were micro-metastasis detected (Group C). Group A had the best prognosis with a biochemical failure-free survival at 10 years of 98%, Group B patients were at risk of late failure, with a similar biochemical failure-free survival to Group A for up to 5 years, while Group C was at high risk for early failure. Overall, Gleason 7 patients had a worse biochemical failure-free survival as compared with Gleason 6 patients, 70% of Gleason 7 patients had MRD detected after RP.

In all groups, Gleason 7 patients had a lower biochemical failure-free survival and a shorter mean time to biochemical failure than Gleason 6 patients, which is consistent with other reported studies, that patients with CPCs detected in non-metastatic disease have a worse prognosis [35, 36].

That Group A Gleason 7 patients had an observed survival of 100% after 5 years of follow-up implies that there is a subgroup of Gleason 7 patients with excellent progress, although representing less than 30% of all Gleason 7 patients in the study group. Similarly, approximately 19% of Gleason 6 patients were classified as Group C, with an observed biochemical failure-free survival of only 17% at 10 years and implies that there is a subgroup of Gleason 6 patients with a worse prognosis.

The study has several limitations which may in part explain this observation; first, we used the pre-2005 Gleason updated classification (we maintained the old Gleason 7 score rather than 3 + 4 and 4 +3) [37]. Also, the low number of patients in some of the subgroups does not permit more detailed analysis.

The clinical reports of patient outcome show heterogeneity in Gleason 6 and Gleason 7 patients that morphological classification, the best predictor of outcome, does not differentiate between biological subtypes of each Gleason score.

Patients with Gleason 3 + 4 have a threefold decreased risk of treatment failure when compared with Gleason 4 + 3 patients, those with favourable Gleason 7 (intermediate risk) have a significantly lower risk of metastasis-free survival and prostate cancer-specific mortality [38]. The authors also reported that favourable risk intermediate prostate cancer had a similar outcome as low-risk prostate cancer, implying the heterogeneity of Gleason 7 tumours. More recently, a prostate cancer genomic classifier identified patients with a high risk of metastasis and death. This measures the biological potential of cancer, rather than morphological features, and out-performed the Gleason score and tumour stage [39]. A 30 gene mRNA expression signature improved predictions of indolent and lethal outcome of men with intermediate-risk Gleason 7 score, independent of whether the Gleason score was 3 + 4 or 4 + 3 [40]. For both Gleason 7 scores, there were indolent and lethal variants of prostate cancer.

Using this simple classification of MRD, which can be determined in the routine histocytochemical laboratory of a general hospital, it was possible to identify prognostic subgroups for each Gleason score. Mitsiades *et al* [41] using RT-PCR to detect CPCs and bone marrow biopsy micro-metastasis prior to surgery, we able to classify the patients into low, intermediate and high-risk groups for biochemical failure.

Independent of possible mechanisms, the study suggests that there are possibly biological differences within Gleason 6 and 7 prostate cancer patients which will determine the outcome after RP for pT2 cancer. Using a simple MRD classification, three different populations of patients can be identified with different relapse characteristics, very low risk of failure and early and late treatment failure. Although this simple system of MRD classification allows risk stratification of prostate cancer patients, the future molecular characterisation of these tumour cells may allow for individualised treatments that are more effective, potentially reveal targets to prevent relapse and avoid overtreatment of patients with indolent MRD. There is a clinical need to delineate the patients with indolent MRD as they present a different biological and thus clinical process, which may require different treatment strategies. The limited number of patients in this study does not permit concrete conclusions and larger prospective studies are required to confirm or refute these results.

## Conclusions

The study suggests that G6 and G7 pT2 patients are heterogeneous and have different patterns of MRD and relapse. Risk stratification using MRD sub-types may help to define the need for adjuvant therapy as well as patients who would not benefit from the immediate treatment. This needs confirmation with large randomised long-term trials.

## Conflicts of interests

Dr Murray reports having received consultancy fees from Viatar CTC Solutions.

## Funding

The study was supported by a Hospital de Carabineros de Chile research grant.

## Figures and Tables

**Table 1.** Clinical-pathological features of the three prognostic groups for 249 men treated by RP for prostate cancer and follow-up time of 10 years for biochemical failure.

Characteristic	Prognostic group			
	A absence of CPC absence of mM *n* = 142	B absence of CPC presence mM *n* = 53	C presence of CPC *n* = 54	*p* value two tail
Age (years) Median; IQR	64; 11	68;13	67.5; 10	0.3013^a^
PSA (ng/mL) Median; IQR	4.9; 1.1	4.9; 2.1	5.3;1.5	0.05 ^a^
Gleason Score 6 *n* (%) 7 *n* (%)	127/197 (64.4) 15/52 (28.9)	37/197 (18.8) 16/52 (30.8)	33/197 (16.8) 21/52 (40.3)	<0.01^b^

IQR = interquartile range; CPC = circulating prostate cell; mM = micro-metastasis; PSA = serum total prostate-specific antigen; ≤ = Equal or less than

a Kruskal–Wallis test

b Pearson’s chi-squared test

**Table 2.** Comparing observed survival (Kaplan–Meier) versus predicted (Model FP) biochemical failure-free progression at 3, 5 and 10 years by three prognostic groups and Gleason score of 7 (≤ 6 and 7) on 249 men treated by RP for prostate cancer.

Variable predictor			3 years		5 years		10 years	
			Survival observed^a^ % (95% CI)	Average survival predicted^b^	Survival observed^a^ % (95% CI)	Average survival predicted^b^	Survival observed^a^ % (95% CI)	Average survival predicted^b^
Prognostic Group	A *n* = 142	Gleason score 6 *n* = 127	100	99.99	100	99.81	97.84 (91.63–99.45)	98.81
		Gleason score of 7 *n* = 15	100	99.60	100	99.20	NO	97.95
	B *n* = 53	Gleason score 6 *n* = 37	100	99.88	100	95.46	90.00 (47.30–98.53)	74.77
		Gleason score of 7 *n* = 16	100	93.17	100	86.77	63.47 (42.06–78.77)	69.70
	C *n* = 54	Gleason score 6 *n* =33	96.97 (80.37–99.57)	93.65	78.79 (60.59–89.27)	77.60	19.03 (7.01–35.50)	21.81
		Gleason score of 7 *n* = 21	38.10 (18.31–57.78)	52.87	14.29 (3.57–32.12)	28.29	4.76 (0.33–19.70)	4.60

%: percentage; CI = confidence interval; ≤ = Equal or less than; NO = not observed

a Observed survival = Kaplan–Meier Survival

b Predicted Survival Model FP = average proportion of mean survival for a given group determined on Flexible Parametric (FP) survival model. The FP model incorporates: age (continuous variable), prognostic group B (dummy variable), prognostic group C (dummy variable) and Gleason score of 7 (dummy variable) with one degrees of freedom for the restricted cubic spline function used for the baseline hazard rate (DF1) and also, consider the dummy variables Gleason score of 7 as variable time-dependent effect using one degree of freedom for its fit in model (DFTVC1).

**Table 3.** RMST at 10 years for biochemical failure determined by using of curves Kaplan–Meier and FP survival model, on 249 men treated by RP for prostate cancer.

Variable predictor			RMST Kaplan–Meier^a^ years (95% CI)	RMST FP Model^b^ years (95% CI)
Prognostic Group	A *n* = 142	Gleason score 6 *n* =127	9.92 (9.80–10.03	9.97 (9.965–9.964
		Gleason of 7 *n* = 15	8.42	9.91 (9.89–9.94)
	B *n* = 53	Gleason score 6 *n* = 37	9.67 (9.05–10.29)	9.26 (9.20–9.33)
		Gleason score of 7 *n* = 16	9.42 (9.09–9.75)	8.62 (8.14–9.1)
	C *n* = 54	Gleason score 6 *n* = 33	7.11 (6.27–7.95)	7.11 (6.91–7.31)
		Gleason of 7 *n* = 21	3.07 (2.23–3.90)	3.79 (3.49–4.08)

% = percentage; CI = confidence interval; ≤ = Equal or less than

a The RMST is the area under the Kaplan–Meier survival curve, determined by the numerical integration

b The RMST is the area under the FP survival model. The FP model incorporates: age (continuous variable), prognostic group B (dummy variable), prognostic group C (dummy variable) and Gleason score of 7 (dummy variable) with one degrees of freedom for the restricted cubic spline function used for the baseline hazard rate (DF1) and also consider the dummy variables Gleason score of 7 as variable time-dependent effect using one degree of freedom for its fit in model (DFTVC1)
